# Impact of drying and cooling rate on the survival of the desiccation-sensitive wheat pollen

**DOI:** 10.1007/s00299-021-02819-w

**Published:** 2022-01-31

**Authors:** Daniela Impe, Daniel Ballesteros, Manuela Nagel

**Affiliations:** 1 Leibniz Institute of Plant Genetics and Crop Plant Research (Leibniz-IPK), Corrensstraße 3, 06466 Seeland OT Gatersleben, Germany; 2 Present Address: Institute of Experimental Botany of the Czech Academy of Science, Rozvojová 263, 165 02 Prague 6, Czech Republic; 3 Royal Botanic Gardens Kew, Wakehurst Place, Ardingly, RH17 6TN UK; 4 Present Address: Universitat de Valencia, Facultad de Farmacia, Av. Vicent Andres Estelles s/n, 46100 Burjassot, Spain

**Keywords:** Cryomicroscopy, Differential scanning calorimetry, Hybrid breeding, Impedance flow cytometry, Pollen viability

## Abstract

**Key message:**

Fast-drying and cooling induce fast intracellular water loss and reduced ice-crystal formation, which may promote the formation of intracellular glasses that might improve the likelihood of wheat pollen survival.

**Abstract:**

Long-term storage of pollen is important for the fertilization of spatially or temporally isolated female parents, especially in hybrid breeding. Wheat pollen is dehydration-sensitive and rapidly loses viability after shedding. To preserve wheat pollen, we hypothesized that fast-drying and cooling rates would increase the rate of intracellular water content (WC) removal, decrease intracellular ice-crystal formation, and increase viability after exposure to ultra-low temperatures. Therefore, we compared slow air-drying with fast-drying (dry air flow) and found significant correlations between pollen WC and viability (r = 0.92, *P* < 0.001); significant differences in WCs after specific drying times; and comparable viabilities after drying to specific WCs. Fast-drying to WCs at which ice melting events were not detected (ΔH = 0 J mg^−1^ DW, < 0.28 mg H_2_O mg^−1^ DW) reduced pollen viability to 1.2 ± 1.0%, but when drying to 0.39 mg H_2_O mg^−1^ DW, some viable pollen was detected (39.4 ± 17.9%). Fast cooling (150 °C min^−1^) of fast-dried pollen to 0.91 ± 0.11 mg H_2_O mg^−1^ DW induced less and a delay of ice-crystal formation during cryomicroscopic-video-recordings compared to slow cooling (1 °C min^−1^), but viability was low (4.5–6.1%) and comparable between cooling rates. Our data support that the combination of fast-drying and cooling rates may enable the survival of wheat pollen likely due to (1) a reduction of the time pollen would be exposed to drying-related deleterious biochemical changes and (2) an inhibition of intracellular ice-crystal formation, but additional research is needed to obtain higher pollen survival after cooling.

**Supplementary Information:**

The online version contains supplementary material available at 10.1007/s00299-021-02819-w.

## Introduction

Preservation of pollen from flowering plants is an important way to complement plant biodiversity conservation efforts and to widen genetic diversity in breeding programs. The storage of haploid male gametophytes allows fertilization of spatially or temporally isolated parents and supports reproduction in the absence of efficient and effective pollinators (Dinato et al. 2020). Wheat (*Triticum aestivum*), the second most produced crop (http://www.fao.org/faostat/), develops desiccation-sensitive pollen which has completed second pollen mitosis and is tricellular before shedding. This pollen type is ready to germinate upon landing on the stigma (Brewbaker 1967; Franchi et al. 2002) and loses its viability when exposed to ambient laboratory conditions for 60 min or to field conditions for 30 min (D´Souza 1970). So far, the time to pollinate the female parents during crossing and hybrid breeding trials is limited to a short time window and only little information has been reported on its desiccation and low-temperature stress tolerance. Therefore, studies on long-term storage of viable wheat pollen would be of high interest to support preservation, accelerate breeding programs, and to promote new breeding options.

Most pollen grains have water contents (WCs) below 30% (fresh weight (FW) basis) at dispersal and are termed desiccation-tolerant, partially dehydrated or orthodox. This pollen type is often small, between 30 μm and 100 μm (Pacini and Franchi 2020), and is able to be stored at low WCs (< 15% FW basis) and low temperatures for longer periods (Dinato et al. 2020). The low WCs do not favour ice-crystal formation and, thus, freeze-injuries during storage at sub-zero temperatures or ultra-low temperatures (< − 150 °C) (Dinato et al. 2020). To achieve low WCs, pollen is often dried until the cytoplasm enters to a metastable solid state known as the glass (Buitink et al. 1996). Drying may be performed naturally at anthesis, or forced at ambient conditions (if relative humidity (RH) < 75%), above salt solutions (Connor and Towill 1993) or silica gel (Walt and Littlejohn 1996). Protocols for dry storage at low or ultra-low temperatures have been employed for pollen of various crops (Hecker et al. 1986; Sacks and Clair 1996; Souza et al. 2018), medicinal plants (Gaudet et al. 2020), woody trees (Alba et al. 2011; Maryam et al. 2017; Zhang et al. 2017), ornamental plants (Geng et al. 2013; Xu et al. 2014), and endangered species (Rajasekharan et al. 2013).

Pollen grains that have WCs above 30% (FW basis) at dispersal, termed desiccation-sensitive, partially hydrated or recalcitrant, are often bigger (15 to 150 μm) (Pacini and Franchi 2020) and hardly tolerate water loss below 30% (FW basis). To store dehydration-sensitive pollen, the levels of WC reached must be carefully balanced, and stay higher to those that generate desiccation damage and lower or close to the limit to those in which water can freeze (Nebot et al. 2021). For desiccation-sensitive pollen of some members of the *Poaceae* family, e.g. maize (*Zea mays*) (Barnabás and Rajki 1976; Nath and Anderson 1975), pearl millet (*Pennisetum glaucum*) (Hanna 1990), protocols for cryogenic storage were successfully developed. The additional application of rapid dehydration by a stream of dry air showed to increase the survival to low WCs and to improve the cryostorage success of embryonic axes of various desiccation-sensitive seeds (Berjak et al. 1993; Pammenter et al. 1991, 1998; Wesley-Smith et al. 2001a, b) and has been applied to pollen of maize (Buitink et al. 1996; Nebot et al. 2021).

Besides drying, pollen viability can be affected by cooling speed, i.e., slow vs. fast cooling, during the cryogenic procedure (Dinato et al. 2020). In general, for cells, tissues, or small organs, slow cooling is often conducted in two steps. After samples are cooled at 0.1 to 5 °C min^−1^ down to − 35 to − 40 °C which is above the ice nucleation temperature, samples are exposed to liquid nitrogen (LN) for long-term storage. Most intracellular water is removed by freeze dehydration. If applied, cryoprotective substances such as dimethyl sulfoxide (DMSO) interact and modulate the distribution of water in- and outside the cell. Fast cooling applies cooling rates of > 100 °C min^−1^ to enable vitrification processes (Wolkers and Oldenhof 2021). Biological systems vitrify when cell viscosity increases, glassy structures are formed, and water molecules are prevented from aggregating into larger ice crystals (Ganeshan et al. 2008). To increase intracellular viscosity, often cells or small organs are exposed to dehydrating agents such as Plant Vitrification Solutions (PVS) which consist of sucrose, glycerol, and DMSO (Fahy and Wowk 2015). Nevertheless, re-crystallization events can occur during warming and affect the viability (Bajaj 1985; Mazur 1984; Meryman and Williams 1985). Therefore, rapid warming by immersing the samples in a warm water bath at 37 to 40 °C for 1 to 5 min is often applied (Ganeshan et al. 2008). The kinetics of intracellular ice formation accompanying cooling and warming can be recorded by advanced video-cryomicroscopy (Karlsson 2015) introduced in 1971 (Diller and Cravalho 1971). This technology has been frequently used in cryopreservation studies of mammalian cells (Scheiwe and Korber 1984; Stott and Karlsson 2009), but its application in plant cryopreservation is still novel. As it provides additional data to assess the risk of cryoinjury (Karlsson 2015), it may guide further advancements in the cryopreservation procedure of, i.e., dehydration-sensitive pollen.

The aim of our study is to investigate systematically the relationship between drying rate, pollen WC, and cooling rates on wheat pollen viability before and after exposure to ultra-low temperatures. Video-cryomicroscopy and differential scanning calorimetry (DSC) were employed to monitor events of ice crystallization in wheat pollen dried at two drying rates, to test the hypotheses that (1) pollen desiccation tolerance increases in fast-dried compared to slow air-dried pollen, (2) ice crystallization can be reduced in fast-dried and fast-cooled desiccation-sensitive pollen, and (3) the higher desiccation tolerance of fast-dried pollen allows a higher survival after rapid cooling. Results obtained are discussed in relation to main reasons for wheat pollen damage during dehydration and cooling, and are used to recommend further protocol improvements for the cryopreservation of wheat pollen.

## Materials and methods

### Plant material and pollen sampling

Seeds of the spring wheat lines TRI 9102 (https://doi.org/10.25642/IPK/GBIS/9074) and TRI 3633 (https://doi.org/10.25642/IPK/GBIS/3633) were provided by the Federal Ex situ Gene Bank of agricultural and horticultural plants at IPK Gatersleben. Seeds of the winter wheat line ‘Ferrum’ (KWS, licensed since 2012) were commercially available. Seeds were germinated in a standard culture medium (Substrate1, Klasmann-Deilmann GmbH, Geeste, Germany) at 20 ± 2 °C. One-week old seedlings were subjected to 4 ± 1 °C for 4 weeks. Vernalized plants were transferred into pots containing a sand/soil mixture (70% compost soil, 20% white peat, 10% sand) and grown under optimum conditions (regular watering and fertilization, 16 h light) at 20 ± 2 °C in the greenhouse.

At the beginning of anthesis, spikes were cut between 8:00 and 10:00 a.m., kept in water and used within 6 h. Only mature pollen was used for all experiments. To stimulate pollen maturation, awns, glumes, and lemmas were carefully removed and pollen was sampled when lodicules swelled, the stigma fanned out, filaments elongated, and anthers enlarged and turned greenish to bright yellow (Impe et al. 2020). Before the tip of the anther opened, at minimum three anthers were taken and pollen shedding was supported by opening gently with a needle. Mature pollen was used immediately after anthesis as a control and termed ‘fresh’. Due to different flowering times, pollen of different wheat lines had to be used for different experiments.

### Pollen treatment and water content

Fresh mature pollen was collected from 5 to 8 anthers of one spike, transferred to a mesh of pore size 30 µm, and fixed with a second layer of mesh in a so-called flash-dryer according to Buitink et al. (1996) and Nebot et al. (2021). The pollen was exposed to a stream of dry air (10.7 ± 0.1% RH) equilibrated above 250 g silica gel at room temperature which reached equilibrated RH 4 min after opening. By this method, pollen was fast-dried for 1, 2, 3, 5, 7, 10, 12, 15, 20, and 60 min and corresponding RHs are given in Figure S1. Pollen air-dried at 60.0 ± 0.1% RH and room temperature (23.0 ± 0.4 °C) for 10, 20, 30, 40, and 60 min was used as a reference of slow-dried pollen, and fresh pollen served as a reference of non-dried pollen.

**Fig. 1:**
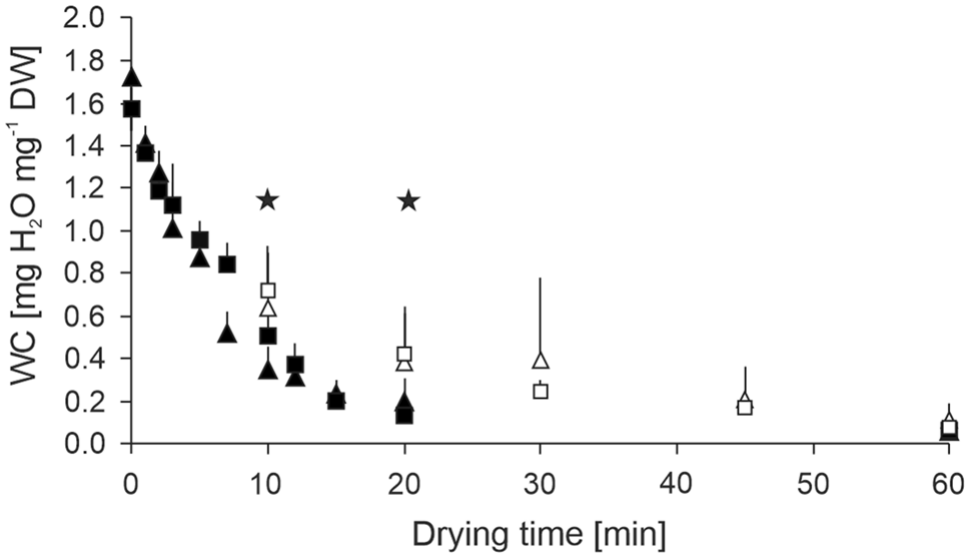
Fast-drying resulted in a faster reduction in pollen water content (WC). Pollen of the wheat lines ‘Ferrum’ (triangles) and TRI 9102 (squares) were slow air-dried (white symbols) or fast-dried (black symbols) at room temperature at low relative humidity for up to 60 min. Mean and standard deviation are shown for each drying time representing for 4–5 replicates each. Stars mark significant differences in WC between fast and air-dried pollen at *P* < 0.05 (comparison made only for available sampling points, hereafter 10, 20, and 60 min of drying). The dashed line indicates WC after 5 min drying time used for following cooling experiments. DW, dry weight

Pollen WC was measured on a set of fresh, fast-dried, and air-dried pollen. Pollen was transferred in an aluminium pan, hermetically sealed, and weighted. To determine pollen WC, pans were perforated and exposed to 100 °C for 24 h. Afterwards, pans were re-weighted and the difference between FW and dry weight (DW) was calculated and expressed as mg H_2_O mg^−1^ DW (1) or percentage of FW (2)

1 $${\text{WC }}\left[ {{\text{mg H}}_{2} {\text{O mg}}^{{ - 1}} {\text{ DW}}^{{ - 1}} } \right] = \frac{{\left( {{\text{Weight}}\;{\text{of}}\;{\text{fresh}}\;{\text{pollen}}\left[ {{\text{mg}}} \right] - {\text{weight}}\;{\text{of}}\;{\text{dry}}\;{\text{pollen}}\left[ {{\text{mg}}} \right]} \right)}}{{{\text{weight}}\;{\text{of}}\;{\text{dry}}\;{\text{pollen}}\left[ {{\text{mg}}} \right]}}$$

2 $${\text{WC }}\left[ \% \right] = \frac{{\left( {{\text{Weight}}\;{\text{of}}\;{\text{fresh}}\;{\text{pollen}}\left[ {{\text{mg}}} \right] - {\text{weight}}\;{\text{of}}\;{\text{dry}}\;{\text{pollen}}\left[ {{\text{mg}}} \right]} \right)}}{{{\text{weight}}\;{\text{of}}\;{\text{fresh}}\;{\text{pollen}}\left[ {{\text{mg}}} \right]}} \times 100.$$

Then WC values were plotted against drying time and drying curves were built for each wheat line and drying method. Drying rates were calculated for the first part of the drying curve (0 to 10 min) in terms of mg H_2_O mg^−1^ DW lost per minute (Ballesteros et al. 2014).

### Pollen viability

Pollen viability was assessed by pollen germination and impedance flow cytometry (termed IFC viability) using at minimum four biological replicates each if not otherwise stated. Pollen was germinated on a solid medium containing 594 mM raffinose, 0.81 mM H_3_BO_3_, 2.04 mM CaCl_2_ at pH 5.8. Pollen tubes exceeding the lengths of the pollen radius were counted manually and expressed as percentage of germination. To determine IFC viability, pollen was transferred into 1 mL IFC buffer (AF6, Amphasys, Lucerne, Switzerland), filtered using 100 µm pore size, and loaded onto a chip of 120 µm channel size. The chip was inserted in the IFC (Ampha Z32, Amphasys, Lucerne, Switzerland) and measurements were carried out at 1 MHz at the default settings for wheat pollen. IFC viability of at minimum 1000 pollen was analysed using AmphaSoft 2.0 version (Amphasys, Lucerne, Switzerland) and given as a percentage.

### Differential scanning calorimetry

Fresh, air-dried and fast-dried pollen of TRI 9102 and ‘Ferrum’ were hermetically sealed in aluminium pans, weighted, and scanned using a DSC-Q2000 (Thermal Analysis Instruments, New Castle, USA) equipped with an LN cooling device. Measurements were carried out between 23 °C and − 150 °C using cooling/warming rates of 10 °C min^−1^. The onset of crystallization and melting events were determined from the intersection between the baseline and a line drawn from the steepest segment of the transition peak using the software Thermal Analysis v. 4.4. (Thermal Analysis Instruments, New Castle, USA). Baselines were determined using an empty aluminium pan as a reference. The enthalpy (Δ*H*) of the transition was determined from the area encompassed by the peak and the baseline (Ballesteros and Walters 2007). Exothermic and endothermic changes are expressed on a DW basis and were plotted against the pollen WC. Results were obtained from at least four replicates each for each drying treatment.

### Cryomicroscopy-video-recordings

Fresh, air- and partially fast-dried pollen were used. Partially fast-dried pollen was dried for 5 min and kept still a high IFC viability (~ 80%) after drying, while the WC decreased to 0.91 ± 0.11 mg H_2_O mg^−1^ DW. Although WC was likely above the levels of unfrozen WC (measured at about 0.20–0.40 mg H_2_O mg^−1^ DW for seeds, fern spores, *Typha latifolia* or maize pollen (Ballesteros and Walters 2007; Buitink et al. 1996)), we presumed that this would reduce the risk of over-drying of pollen (< 40% IFC viability) which may occur when pollen is dried for 10 min, down to the unfrozen WC. For comparison, we also used fresh pollen and pollen dried for 60 min (WC < 0.06 mg H_2_O mg^−1^ DW), which was at WC below the unfrozen WC (< 0.26 mg H_2_O mg^−1^ DW), but was completely dead. Pollen from different treatments were dusted onto glass plates and placed in the cryomicroscopic stage system (BCS196, Linkam Scientific Instruments, Tadworth, United Kingdom) which was mounted on a light microscope (Eclipse LV100, Nikon, Tokyo, Japan). The stage was connected to a T95-PE temperature control unit (Linkam Scientific Instruments, Tadworth, UK) and a Dewar vessel containing LN. The temperature program and flow rates were controlled using LinkSys32 software (Linkam Scientific Instruments, Tadworth, UK). Pollen were exposed to slow (1 °C min^−1^) and fast cooling/warming (150 °C min^−1^) in a range between 0 to −40 °C. Details are provided in Table 1. Due to the high rates during fast cooling/warming, the means and standard deviations of temperatures for structural changes of pollen (i.e., darkening, pollen turn black and thawing) were interpolated based on temperature at the start, within the holding phase and at the end of the cooling program and a few temperature points in between taken at the time points when structural changes were visible.

**Table 1:** Cooling/warming programs used to monitor wheat pollen during cryogenic treatments

Temperature program	Setting for video recording	Ramp	Temperature [°C]	Flow rate [°C min^−1^]	Holding time [min]
Slow cooling	Time-lapse video 1 frame per 5 s	1 2 3 4 5	20 0 − 40 0 20	100 1 1 1 100	2
Fast cooling	Real-time video 30 frames per sec	1 2 3	20 − 40 20	150 150 150	3

Time-lapse and real-time videos recorded with a camera attached to the optical output of the microscope were used to monitor structural changes during cooling/warming. To evaluate significant events, NIS software (v. 4.11, Nikon Metrology, Brighton, USA) allowed the extraction of specific images for an exact time point. Three-to-six replicates for each of the six treatments including two cooling/warming programs and three drying treatments were prepared.

### Statistical analysis

Means and standard deviations (SD) were calculated in Excel 365 (Microsoft, Richmond, CA, USA) and data are shown as mean ± SD. Statistical analyses were performed based on unpaired Student’s *t* test. *P* values < 0.05 were considered significant; in the figures, *, **, and *** indicate significant differences at the 0.05, 0.01, and 0.001 levels of confidence, respectively, while no label indicates no significance. Using GenStat 19.1 (VSN International Ltd., 2016), Pearson correlation analysis was performed to find significant relationships at *P* < 0.05 between drying method, WC and viability. To compare the drying rates, slopes of WCs were estimated by linear regression within the first 10 min and 20 min of drying. WC limits for pollen viability and germination were calculated using the effective dose function (probit analysis) in Genstat 19.1 (VSN International Ltd., 2016). Effective dose of WC was estimated at 60% IFC viability and 20% pollen germination.

## Results

### The viability of wheat pollen is affected by the drying rate and water content

Immediately after shedding, pollen of the wheat lines ‘Ferrum’ and TRI 9102 had a steady high WC of 1.72 ± 0.01 mg H_2_O mg^−1^ DW (63.3% of FW) and 1.57 ± 0.10 mg H_2_O mg^−1^ DW (61.1% of FW), respectively. After 10 min of drying, air-dried pollen of ‘Ferrum’ and TRI 9102 lost on average 62.9% (0.64 ± 0.72 mg H_2_O mg^−1^ DW) and 54.2% (0.72 ± 0.20 mg H_2_O mg^−1^ DW) of the FW, respectively. This slow-drying method resulted in a wide range of pollen WCs across replicates as shown by the standard deviation, indicating an inhomogeneous drying of the pollen. The pollen grains dried on a plate, so there was probably passive drying on the surface of the pollen grains exposed to drying air at 60% RH. In comparison, fast-drying resulted in an averaged loss of 79.7% (0.32 ± 0.15 mg H_2_O mg^−1^ DW) and 67.7% (0.51 ± 0.13 mg H_2_O mg^−1^ DW) of FW, respectively (Fig. 1), with more homogenous drying including smaller standard deviation observed. Fast-drying was carried out on pollen “floating” on a stream of 10% RH dry air, which likely dried the whole grain and not just the exposed area. The loss of water during the first 10 min followed a near-linear trend (Fig. 1) and the drying rates (slopes of linear regression curves) were significantly different between the drying approaches (*P* ≤ 0.001) but not between the wheat lines (*P* = 0.133). Fast-dried pollen lost − 0.116 ± 0.080 mg H_2_O min^−1^, while air-dried pollen lost −0.097 ± 0.086 mg H_2_O min^−1^. Overall, fast-drying led to a homogenous, significant (*P* ≤ 0.01) and 1.8- and 1.4-times larger loss of water of ‘Ferrum’ and TRI 9102, respectively, compared to slow air-drying after 10 min. Comparable significant differences (*P* ≤ 0.001) were also found after 20 min of drying (Fig. 1). However, after 60 min, no significant differences were found in the WC of pollen from both lines exposed to air- and fast-drying, which reached steady WC values of 0.08 ± 0.04 mg H_2_O mg^−1^ DW and 0.06 ± 0.02 mg H_2_O mg^−1^ DW, respectively (about 95.2% WC loss from FW). In summary, the flow of dry air during fast-drying caused a larger, faster, and more homogeneous water loss on average compared to slow air-drying, particularly in the first 20 min.

**Fig. 2:**
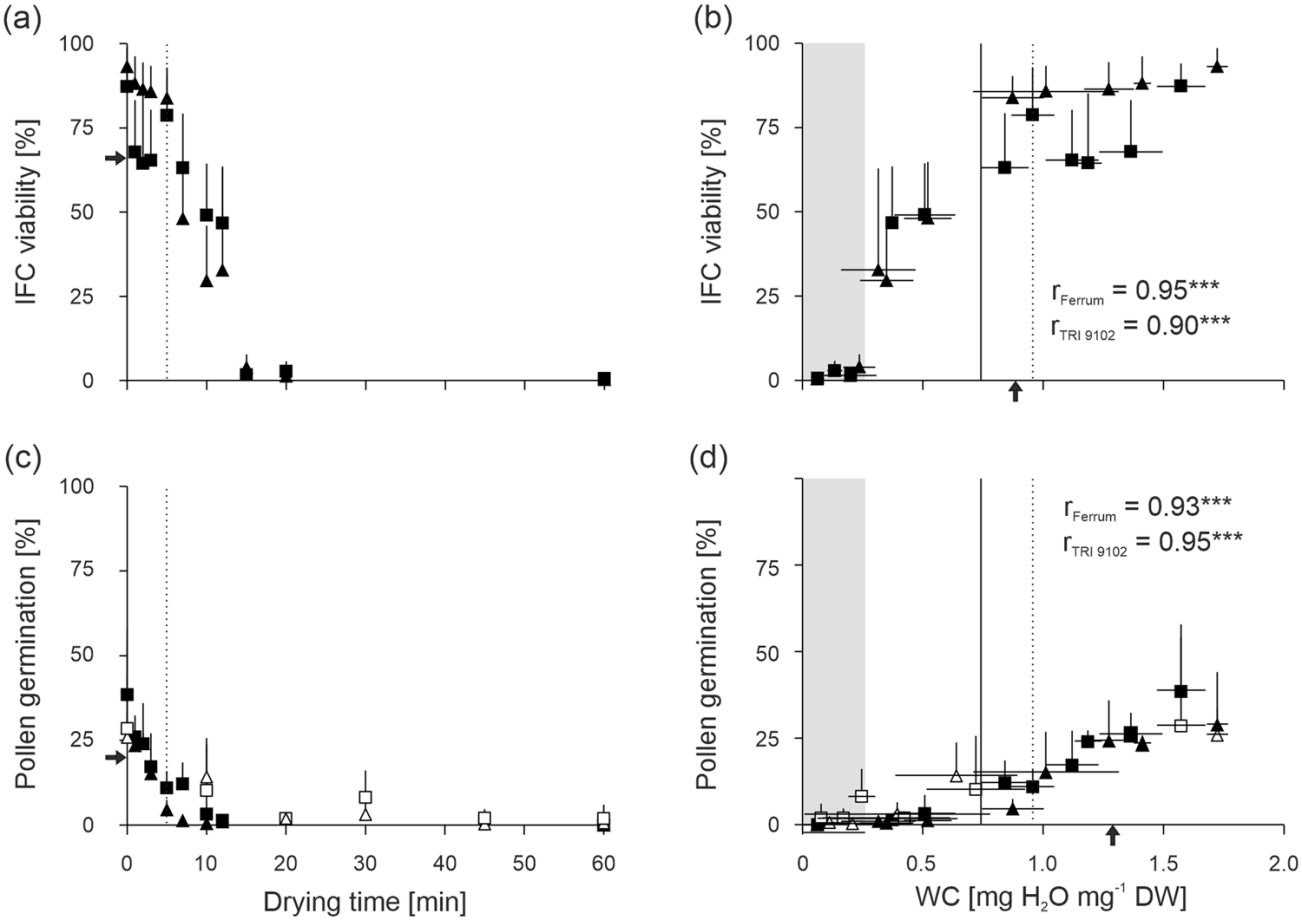
Reduction in pollen water content (WC) affects pollen viability. Pollen of wheat lines ‘Ferrum’ (triangles) and TRI 9102 (squares) were slow air-dried (white symbols) or fast-dried (black symbols) for 1 to 60 min. **a**, **c** Drying time and **b**, **d** pollen WC were plotted against (**a**, **b**) pollen viability determined by impedance flow cytometry (IFC viability) and **c**, **d** pollen germination determined on a raffinose-based medium. Mean and standard deviation are shown for each drying time and respective WCs representing 4–5 replicates each. The dashed lines indicate pollen viability/germination and WCs after 5 min drying time used for following cooling experiments. Shaded areas show WCs at which no frozen water was detected. Solid lines indicate a transition of onset/peak temperatures in the melting curves (compare with Fig. 3b). Black arrows indicate damaging WC (**b**, **d**), at which IFC viability and pollen germination dropped by one-third (**a**, **c**). DW, dry weight

Viability of fresh pollen determined by IFC (IFC viability) was high, 93.2 ± 5.5% and 87.3 ± 6.7% for ‘Ferrum’ and TRI 9102, respectively (Fig. 2a). Pollen germination evaluated on raffinose-based media was lower, ranged between 28.8 ± 15.3% and 38.5 ± 19.3%, respectively (Fig. 2c). After 5 min of fast-drying (WC_‘Ferrum’_ = 0.87 ± 0.13 and WC_TRI9102_ = 0.96 ± 0.09 mg H_2_O mg^−1^ DW), IFC viability was 83.9 ± 6.4% and 78.8 ± 14.2% and pollen germination was 4.5 ± 0.9% and 10.9 ± 1.0% for ‘Ferrum’ and TRI 9102, respectively. After 10 min of fast-drying (WC_‘Ferrum’_ = 0.35 ± 0.11 and WC_TRI9102_ = 0.51 ± 0.13 mg H_2_O mg^−1^ DW), IFC viability decreased further to 29.6 ± 16.3% and 49.1 ± 15.3% for ‘Ferrum’ and TRI 9102, respectively, and pollen germination to 1.9 ± 3.9% for both wheat lines. For comparison, after 10 min of air-drying, pollen germination of both wheat lines dropped to 12.2 ± 12.3% (Fig. 2c), but in this case, WC was significantly higher (0.68 ± 0.22 mg H_2_O mg^−1^ DW). After 60 min of air- and fast-drying, when WC was below 0.08 ± 0.04 and 0.06 ± 0.02 mg H_2_O mg^−1^ DW, respectively, both pollen germination and IFC viability dropped to 0% (Fig. 2c,d), although after 20 min of drying, viability was near zero in both drying regimes. The decrease of viability during drying period (or the reduction of pollen WC) followed a sigmoidal trend (Fig. 2a,b). To estimate the “damaging” WC during drying, we assumed that at this point the IFC viability dropped by one-third to 66% and calculated the WCs using probit transformation. Finally, the damaging WCs were estimated at 0.83 ± 0.11 and 0.94 ± 0.11 mg H_2_O mg^−1^ DW for ‘Ferrum’ and TRI 9102, respectively, during fast-drying. When pollen germination dropped by one-third, at about 20%, the damaging WCs was at 1.29 ± 0.16 and 1.29 ± 0.13 mg H_2_O mg^−1^ DW for air- and for fast-drying, respectively, indicating that damaging WCs were similar between both drying methods and wheat lines. Coefficients of correlation between WC and IFC viability or pollen germination were 0.92 (*P* < 0.001) and 0.94 (*P* < 0.001), respectively, suggesting that pollen viability is tightly linked to WC.

### Thermo-physical properties of wheat pollen are altered by drying procedures before ultra-low freezing

**Fig. 3:**
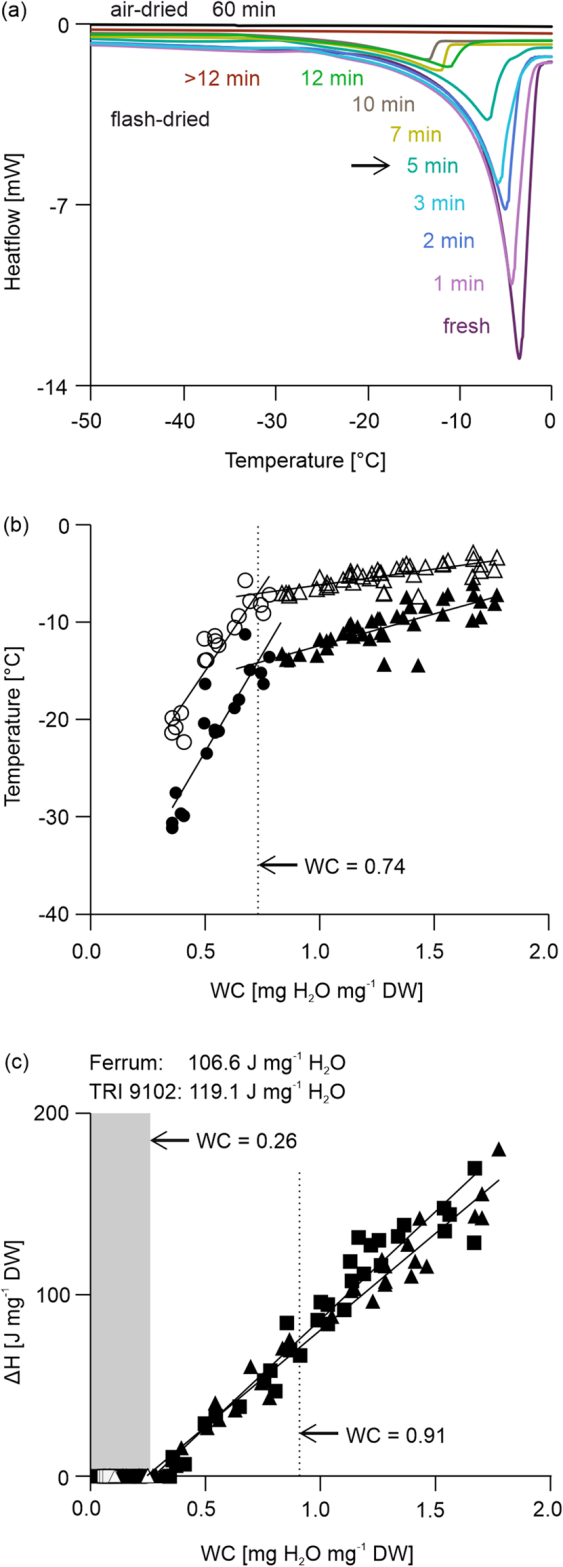
Heat flow decreases when pollen water content (WC) declines and presumably causes fewer crystallization events. Pollen of wheat lines ‘Ferrum’ (triangles) and TRI 9102 (squares) were slow air- (white symbols) and fast-dried (black symbols) for up to 60 min and heat flow measured using Differential Scanning Calorimetry (DSC). **a** Heating thermograms represent warming curves of selected replicates of pollen from wheat line ‘Ferrum’. **b** Relationship between onset temperature (closed symbols) and peak temperatures (open symbols) of the melting transitions with WC. The dashed line indicates the WC (corresponding to > 5 min partially fast-drying time) from which we observed a steep viability decline (compare Fig. 2a, c). **c** Relationship between the pollen WC and the enthalpy (*ΔH*) calculated on basis of the area covered by the warming curves is shown for air- and fast-dried pollen. The dashed line indicates the WC and respective enthalpy after 5 min drying time used for following cooling experiments. Shaded areas show WCs at which no frozen water was detected for both wheat lines and drying methods

Differential scanning calorimetry detected diverse first- and second-order phase transitions in both the cooling and warming scans of samples with varying WCs. During cooling scans, broad exothermic events (crystallization) were detected between − 12 and − 56 °C for wheat pollen having WC between 0.27 and 1.77 mg H_2_O mg^−1^ DW. In some cooling thermograms, multiple broad crystallization peaks were observed in pollen samples dried to a maximum of 5 min (WC between 0.91 and 1.77 mg H_2_O mg^−1^ DW). Additionally, a second small peak, presumably eutectic formations (Sun 2021), was found at ~ − 100 °C and ‘crystallization loops’ appeared in most pollen samples with high WC (> 1 mg H_2_O mg^−1^ DW corresponding to < 5 min fast-drying) (Supplemental figure S2). Broad endothermic events (melting transitions) during the warming program were observed between − 3 to − 32 °C. When the WC of fresh pollen of both wheat lines (1.64 ± 0.11 mg H_2_O mg^−1^ DW) was reduced by fast-drying for up to 15 min (≥ 0.22 ± 0.05 mg H_2_O mg^−1^ DW) or by air-drying for up to 60 min (0.08 ± 0.02 mg H_2_O mg^−1^ DW), the size of the broad crystallization and melting peaks (measured by the enthalpy [*ΔH*] of transition) declined, indicating that these phase transitions are ice-crystal formation and melting events (Fig. 3a).

By plotting WC against onset and peak temperature (Fig. 3b), a moderate decrease of the regression line was shown until WC dropped to 0.74 mg H_2_O mg^−1^ DW. This would be expected for a dilute solution of increasing concentration and was observed in embryos of recalcitrant seeds (Pammenter et al. 1991). Below, a rapid decrease in the onset and peak temperatures of the water melting transitions with WC was observed, indicating a change in the water properties of the cells as the solute concentration continues to increase which strongly affects the melting activity of the ice crystals. Furthermore, the *ΔH* of melting transitions dropped linearly (Fig. 3c) and reached 0 J mg^−1^ DW at < 0.28 mg H_2_O mg^−1^ DW after more than 12 min fast-drying. The value for *ΔH* of melting (slope of linear regression) was 106.6 and 119.1 J mg^−1^ H_2_O for ‘Ferrum’ and TRI 9102, respectively (Fig. 3c). The amount of water that did not freeze was calculated from the intersection of the X-axis and the sloped line drawn for water transitions. Unfrozen WCs were achieved at 0.24 and 0.28 mg H_2_O mg^−1^ DW for ‘Ferrum’ and TRI 9102, respectively. Coefficients of correlation of the regression lines constructed to calculate the unfrozen WC showed high-quality fit for ‘Ferrum’ (*r*^2^ = 0.97) and TRI 9102 (*r*^2^ = 0.95). At WCs below the unfrozen WC pollen showed very low or no viability (Fig. 2b), however, at WCs near the unfrozen WC, some pollen was still viable (39.8 ± 24.8% IFC viability at < 0.39 mg H_2_O mg^−1^ DW corresponding to 12 min drying time), indicating that under these conditions some viable pollen could be exposed to sub-zero temperatures without a lower risk of ice-crystal formation. However, to avoid over-drying of pollen when drying to WCs below the unfrozen WC, in further freezing experiments, we applied partially fast-drying for 5 min which provided > 50% IFC viability.

### Partial fast-drying and fast cooling/warming may contribute to pollen survival by reducing intracellular ice-crystal formation

**Fig. 4:**
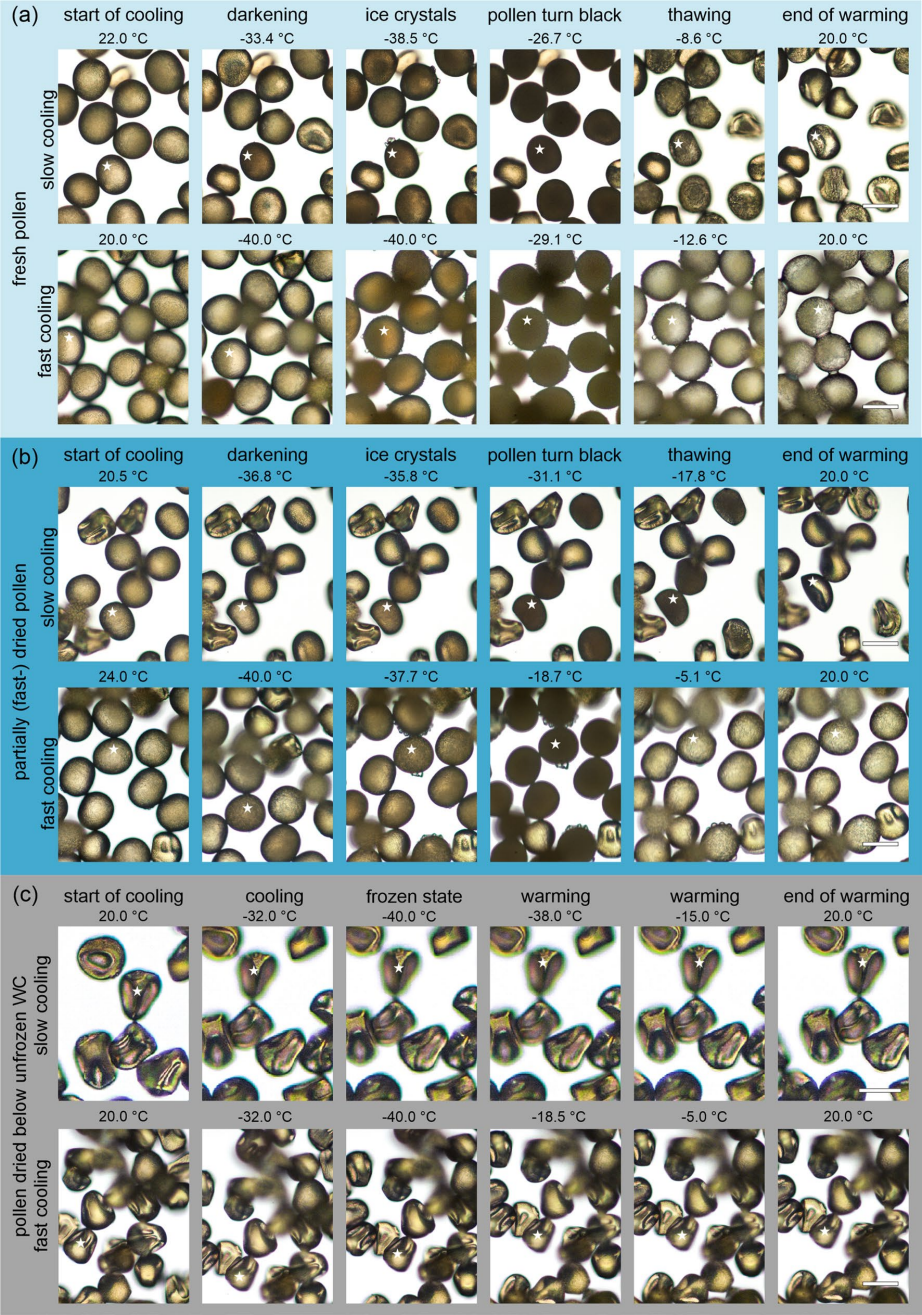
Slow and fast cooling/warming combined with various drying treatments infers a variation in the structural changes in wheat pollen that occur at different temperatures. **a** Fresh pollen (light blue panel), **b** pollen partially fast-dried for 5 min (dark blue panel), and **c** pollen air-dried to WCs below the unfrozen WC for 60 min (grey panel) of wheat line TRI 3633 were cooled and warmed at 1 °C min^−1^ (slow cooling) and 150 °C min^−1^ (fast cooling). Significant events (i.e., darkening, surface ice crystallization, pollen turn black, and thawing) were visualized by extracting images from the videos in Supplementary video S1 to S6; the range of temperatures is shown in Fig. 5. Due to movements in the Linkam chamber, for better orientation, we marked the same pollen in each series (row) with a star. Scale bars indicate 50 μm, size and magnification were identical for all images

**Fig. 5:**
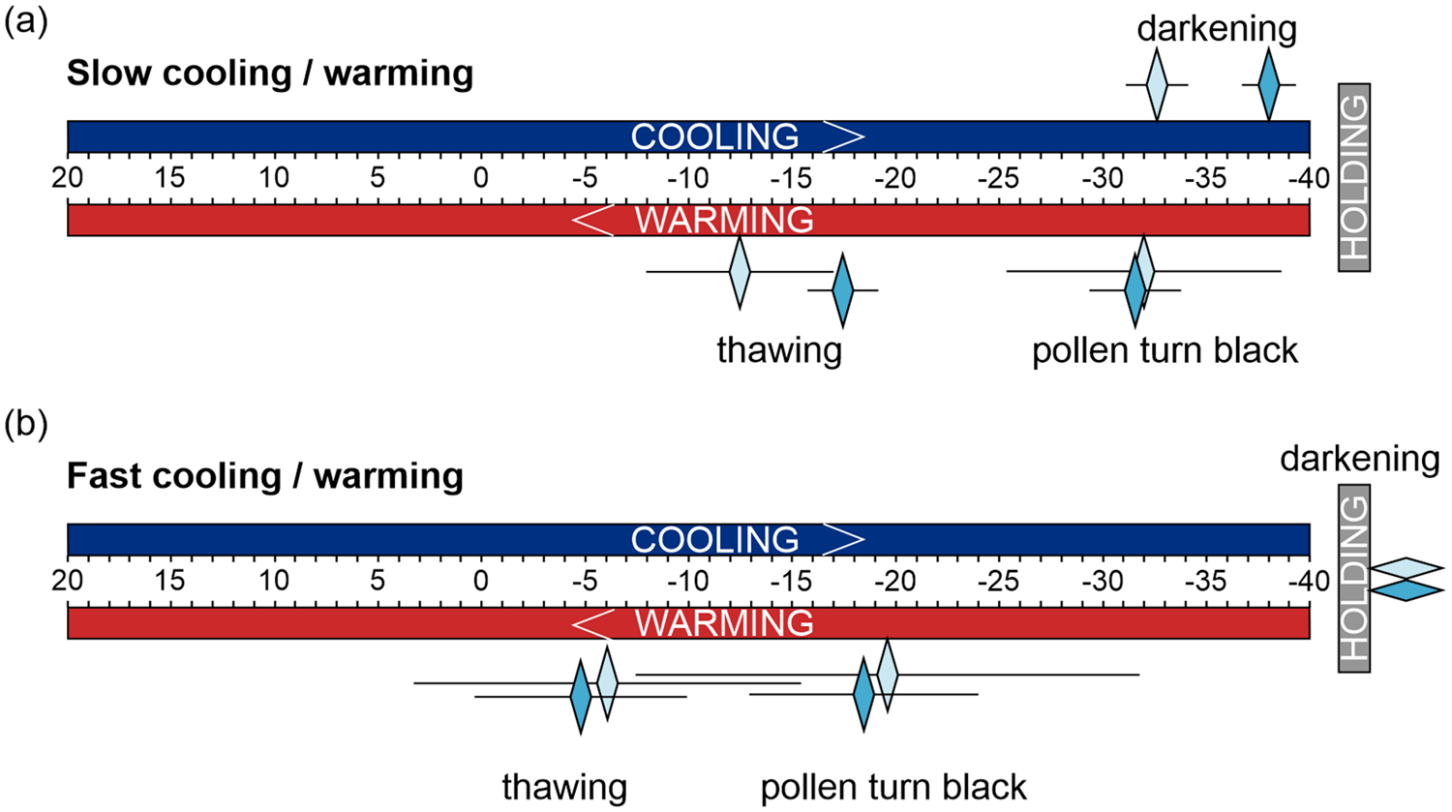
Cooling/warming rate and pollen water content affect crystallization and melting events. Fresh pollen (light blue diamonds) and pollen partially fast-dried for 5 min (petrol blue diamonds) of the wheat line TRI 3633 were used to evaluate the significant events during **a** slow cooling/warming at 1 °C min^−1^ and **b** fast cooling/warming at 150 °C min^−1^. Temperature ranges (diamonds and lines) are given for significant structural changes of the pollen (darkening, pollen turn black, thawing) exposed to different cooling/warming approaches. Diamonds and horizontal lines represent means and standard deviations of 5 biological replicates for each condition. For fast cooling/warming and due to the high cooling rate, means and standard deviations were interpolated based on temperature at the start, within the holding phase, and at the end of the cooling/warming program and a few temperature points in between

**Fig. 6:**
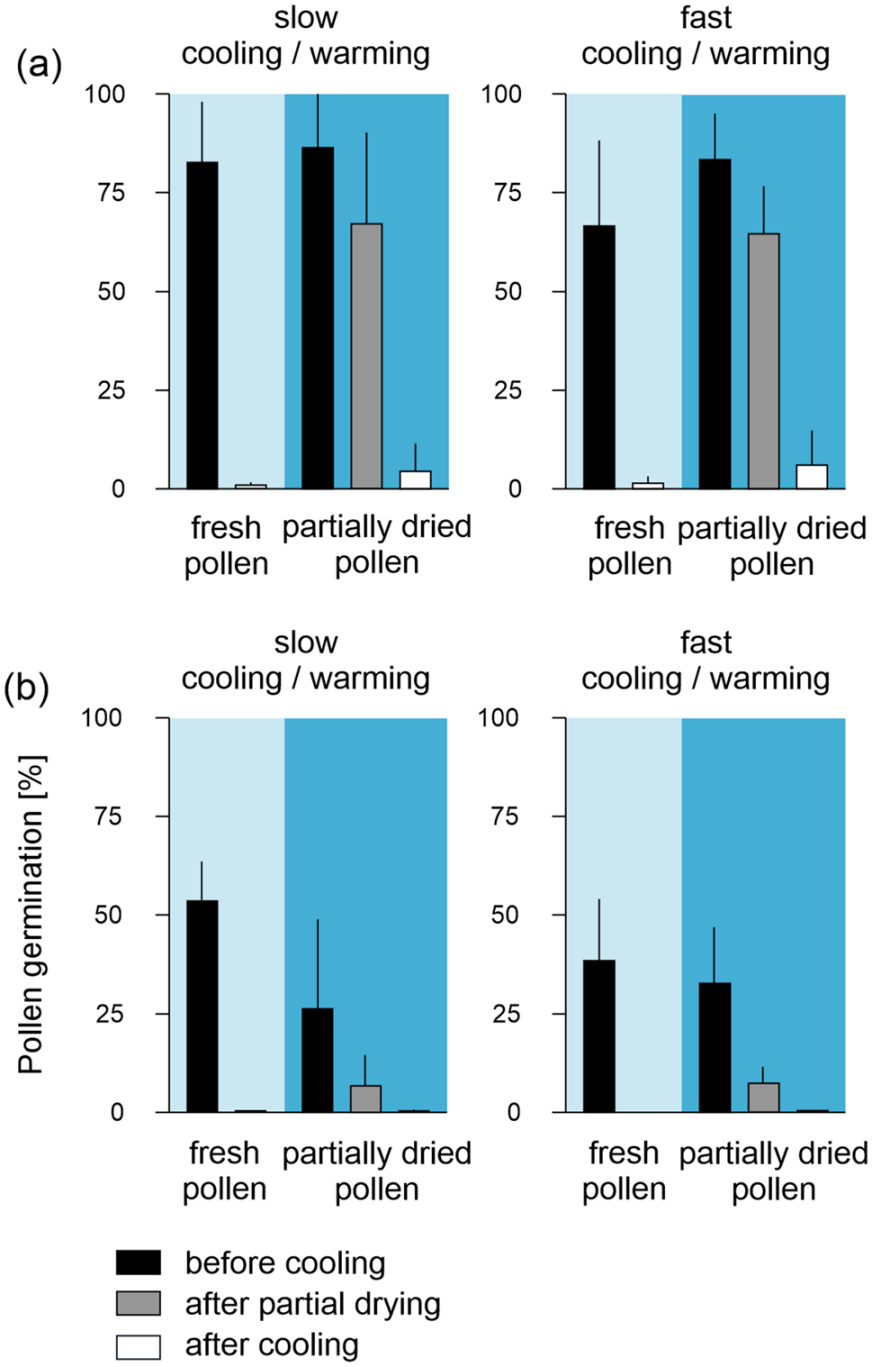
Partial fast-drying increases the chance of pollen survival after cooling/warming. Fresh pollen (black bars), and pollen fast-dried for 5 min (grey bars) of the wheat line TRI 3633 was exposed to slow cooling/warming at 1 °C min^−1^ and fast cooling/warming at 150 °C min^−1^. Pollen viability was analysed by **a** Impedance Flow Cytometry (IFC viability) and **b** as pollen germination on a raffinose-based media before (black bars), after fast-drying (grey bars) and after cooling (white bars). Bars and lines show means and standard deviations of 5 biological replicates

Changes in structure and colour were observed in fresh and partially fast-dried pollen (with a WC above the unfrozen WC) but not in fully dried pollen (to WCs below the unfrozen WC), during both slow and fast cooling/warming. During slow cooling, both fresh and partially fast-dried pollen suddenly ‘flashed’ and turned dark at − 31.6 ± 1.0 °C and − 38.0 ± 0.8 °C, respectively. The so-called ‘darkening’ was followed by the formation of ice crystal on the surface of the pollen grains (Figs. 4a, b, 5a, Supplemental video 1–2). During slow warming, pollen turned completely black at − 32.4 ± 6.1 °C and − 31.5 ± 1.7 °C, respectively, and thawing of ice crystals began at − 12.6 ± 4.0 °C for fresh pollen and at − 17.6 ± 1.2 °C for partially fast-dried pollen. At the end of the program, fresh pollen shrunk massively and had an IFC viability of 0.9 ± 0.7%, while the partially fast-dried pollen also shrunk, but had an IFC viability of 4.5 ± 7.0%. During fast cooling, less and/or smaller ice crystals were observed around the pollen. Darkening occurred in both fresh and partially fast-dried pollen, when temperature was hold at − 40 °C for 3 min and turned completely black at − 19.6 ± 11.6 °C and − 18.6 ± 5.0 °C during fast warming, respectively (Fig. 4a,b, 5b, Supplemental video 3–4). At the end of the fast warming step, fresh pollen and partially fast-dried pollen appeared rounder were less dehydrated and showed some viability (but not germination) (fresh pollen: 1.5 ± 1.7%, partially fast-dried pollen: 6.1 ± 8.8%) compared to slow cooling/warming (Fig. 6). In addition, the change in colour and thawing of ice crystals (fresh pollen: − 6.0 ± 8.8 °C, partially fast-dried pollen: − 4.9 ± 4.6 °C) occurred at about 10 °C higher temperatures compared with slow cooling/warming (Fig. 5b). Pollen dried below the unfrozen WC had an IFC viability of 0% (Fig. 2) and showed no darkening or ice-crystal formation at either slow or fast cooling (Fig. 4c, Supplemental video 5–6). Overall, pollen treated with a combination of partially fast-drying and fast cooling/warming tend to have visually less and/or smaller ice crystals that may support pollen survival.

## Discussion

The preservation of wheat pollen is important for conventional breeding, hybrid breeding and production and the conservation of wheat genetic diversity. Currently, any prolonged storage, more than several hours, is not possible due to the high desiccation sensitivity of mature pollen after shedding. In this paper, we investigated how drying rate and cooling/warming rate affect pollen viability and the physicochemical properties of the water in their cells. We found that viability in pollen was lost soon during the drying process, even before any changes in the structural properties of water are detected by DSC. However, about 40% of wheat pollen retained viability at WCs > 0.28 mg H_2_O mg^−1^ DW, a WC slightly above the limit at which ice freezing/melting events occur. At this WC, pollen grains appear to have highly concentrated cytoplasm based on the thermal responses of the freezing water, which open the option for the development of successful ultra-low-temperature storage. We also measured that the combination of fast-drying, fast cooling, and warming rates during video-cryomicroscopy resulted in a reduction and delay of ice-crystal formation, a reduction of the temperature for intracellular water crystallization, and an increase in the temperature of intracellular ice melting. All three properties may favour the formation of intracellular glasses and could contribute to the success of wheat pollen cryopreservation; however, a fine-tuning of the combination of fast partial-drying and cooling rates is needed to achieve higher survival/germination rates.

### Desiccation damages prior cooling can be reduced by rapid drying

Dependent on the drying rate and the amount of water removed, different types of damages compromise survival in desiccation-sensitive pollen and tissues (Pammenter and Berjak 2014). At high WC, dehydration causes physical damages by reducing cell volume (Pammenter and Berjak 2014). In wheat, we observed some shrinkage of the pollen grains when the pollen population was dried to 0.91 ± 0.11 mg H_2_O mg^−1^ DW (Fig. 4b). Further intense drying causes “desiccation damage sensu stricto” (Walters et al. 2001). The WC at which 33% of the initial wheat pollen viability is lost was estimated at between 0.83 ± 0.11 and 1.29 ± 0.16 mg H_2_O mg^−1^ DW depending on the viability testing method used. We considered these WC ranges to be an indication of the WC limit at which the pollen was significantly damaged and refer to them as damaging WC. Compared to embryonic axes of recalcitrant seeds that retain high viability (over 90%) upon drying to near freezable WCs at ~ 0.25–0.50 mg H_2_O mg^−1^ DW (Wesley-Smith et al. 2001a, b), the damaging WC limit determined for wheat pollen in the current study was very high. By contrast, maize pollen dried to 0.55 mg H_2_O mg^−1^ DW can still retain over 75% germination (Buitink et al. 1996). In wheat pollen, the damaging WC determined fell above the break in the relation between WC and water melting peak/onset temperature (Fig. 3b) detected at 0.74 mg H_2_O mg^−1^ DW. Although conjectural, this break might be due to a change in the water properties of the cytoplasm that could be related to a threshold in the concentration of solutes during drying that alter the physicochemical properties of ice-crystal formation (Berjak et al. 1993) and may lead to deleterious molecular interactions (Oliver et al. 2020). When WC is reduced below a critical value, the removal of water from macromolecular and membrane surfaces causes metabolism-linked damages (Pammenter and Berjak 2000). In wheat, pollen lost most of its initial viability around freezable WC at < 0.28 mg H_2_O mg^−1^ DW, ~ 13% FW basis. Extensive membrane damages were found in desiccation-sensitive pollen of *Pennisetum typhoides* and maize dried to 3% and 7 to 8% WC, respectively (Hoekstra et al. 1989; Kerhoas et al. 1987). In wheat pollen, at WC below 0.06 mg H_2_O mg^−1^ DW (2.4%), viability was completely lost and accompanied with the strong shrinkage of wheat pollen (Fig. 4c). At this stage, cells, organelles, and membranes may collapse (Buitink et al. 1998) resulting in cell/pollen death (Bajaj 1985; Pammenter and Berjak 2000; Pammenter et al. 2003).

However, drying damage is considered to be avoided depending on the drying rate. When drying is conducted slowly, electron transfer in mitochondria and plastids is impaired that facilitates the formation of reactive oxygen species (ROS) (Halliwell and Gutteridge 2015; Hendry et al. 1992; Smirnoff 1993) reacting with proteins, lipids, and nucleic acids (Halliwell and Gutteridge 2015). Additionally, the reduced fluidity of the cytoplasm at intermediate WC limits the efficiency of corresponding detoxifying processes (Bailly 2004; Berjak 2006; Vertucci and Farrant 1995). When drying is conducted sufficiently rapid, i.e., by a stream of dry air (Buitink et al. 1996), and WC in dehydration-sensitive cells remain above the critical points, the time in which cells pass through intermediate WC ranges is shorter. Thus, fast-drying may reduce the overall occurrence of metabolism-derived damages (Farrant et al. 1993; Kioko et al. 1998; Pammenter and Berjak 1999; Pammenter et al. 2002; Pritchard and Manger 1998). However, despite the faster and more homogeneous drying induced by the fast-drying method applied in this work (Fig. 1), we found that both the damaging WC calculated by the viability/WC relations (Fig. 2) or the viability loss observed at given WCs was comparable for the air- and fast-drying methods used, suggesting that the different drying rates applied in this study were not sufficient to detect drying-related differences in viability prior to the cooling stage. Nonetheless, fast-drying reduces the time the pollen is exposed to stressful metabolic processes. Due to the additional stress on the pollen by cooling, we think that fast-drying prior cooling increases the chances that the desiccation-sensitive wheat pollen will survive the whole cryopreservation process.

### Wheat pollen can partly survive the loss of freezable water

When cells lose the unbound, freezable water, intracellular viscosity increases (Hoekstra et al. 2001). Upon further drying and/or cooling, the fluid cytoplasm develops properties of a solid structure (Ballesteros and Walters 2007, 2011; Buitink and Leprince 2004) which is non-crystalline (unlike the intracellular ice that forms in the presence of freezable water) and is referred to as a glass. A glass is an amorphous solid that combines properties of a solid and a liquid without any defined structures (Buitink and Leprince 2004). The increase in cytoplasmic viscosity correlates with decreases in molecular mobility (Ballesteros and Walters 2011; Leprince and Hoekstra 1998; Sun 2000) and metabolic activity where most chemical reactions are ceased (Benson 2008). The extremely high cytoplasmic viscosity of the glass and the binding of most water molecules to macromolecules prevent the formation of ice crystals during cooling (Benson 2004). For diverse pollen, ice crystallization events are not detected by DSC typically at WC < 20% (0.21–0.26 mg H_2_O mg^−1^ DW). Below this WC, cryo-injuries can be prevented during freezing and thawing (Dinato et al. 2020). For wheat pollen, we demonstrated that depending on the drying rate, pollen reduced the fraction of freezable water in less than 30 min, but viability was severely impaired, when pollen dried to the freezable WC at < 0.28 mg H_2_O mg^−1^ DW, ~ 13% FW basis. However, slightly above the freezable WC limit, wheat partially fast-dried pollen retained IFC viability at 39.8 ± 24.8%. Although both, desiccation-sensitive and -tolerant, plant tissues are capable of forming glasses upon drying and/or cooling (Buitink et al. 1996), most dehydration-sensitive plant material cannot survive the low WC at which glasses typically form before exposing the samples to sub-zero temperatures (Berjak and Pammenter 2004; Berjak and Pammenter 2004). Nevertheless, cryopreservation was successfully implemented for desiccation-sensitive maize pollen. Dependent on genotype, some varieties retained high viability and seed set after drying to between 12 and 20% WC corresponding to 0.14 and 0.25 mg H_2_O mg^−1^ DW (Barnabás and Rajki 1976, 1981). Inagaki and Mujeeb-Kazi (1994) achieved about 22.1% of maize pollen germination after storage at – 80 °C and 9.5% WC for 4 weeks which was likely due to the removal of the freezable water fraction and the formation of a glassy state below − 25 °C at such WCs (Buitink et al. 1996). To cryopreserve desiccation-sensitive wheat pollen with high viability, existing procedures (Nebot et al. 2021) must be modified to favour the formation of a glassy state while overcoming the constraints of the low viability when WC is reduced below the freezable WC limit. This balance between vitrification without ice formation may be achieved by modulating cooling rates at the WCs at which wheat pollen still retains high viability.

### Fast cooling limits and delays ice-crystal formation compared to slow cooling

The survival of plant cells after freezing is dependent on the lethal effects of intracellular ice. Ice crystals affect the organization of the cytoplasm and damage the membranes of cell organs by increasing their volume (Wolkers and Oldenhof 2021). The formation of intracellular ice can be effectively minimized by the cooling rate (Mazur 2004). At very slow cooling rates (~ 0.1 to 5 °C min^−1^), ice is formed intercellularly. The chemical potential between cells inside and outside leads to water efflux, cell dehydration (Wolkers and Oldenhof 2021), and eventually vitrification of the cytoplasm. However, the dehydration during slow cooling may severely damage desiccation-sensitive cells (Wesley-Smith et al. 2001a, b). When the cooling rate increases (~ 100 °C min^−1^), the time for the water to flow out the cell is not sufficient to prevent supercooling, spontaneous intracellular freezing, and the aggregation of larger ice crystals (Mazur 1984). At very high cooling rates (> 1000 °C min^−1^), more, smaller and evenly distributed ice crystals appear (Dumont et al. 2003) and might be more beneficial for desiccation-sensitive cells compared to dehydration at slower cooling rates. Therefore, in desiccation-sensitive embryonic axes of *Aesculus hippocastanum* L., a combination of fast-drying followed by cooling at very high rates reduced deteriorative processes and enabled the survival of the tissue (Wesley-Smith et al. 2001a, b). When wheat pollen was fast-dried for 5 min and rapidly cooled and warmed in the Linkam chamber using 150 °C min^−1^ (Fig. 3, Supplemental videos 2,3), we observed a reduction of intracellular ice crystals formation and the limitation of the ice-crystal formation to the isothermal step at − 40 °C. Under these conditions, some wheat pollen still showed IFC viability of 6.1 ± 8.8%. To observe processes of ice formation in wheat pollen by cryomicroscopy, we were limited to the rapid cooling rates at max 150 °C min^−1^ and an experimental design conceived to study ice formation in the first stages of cooling. However, higher very rapid cooling rates to < − 130 °C temperatures should be applied in future experiments to combine the effects of fast-drying, evenly distributed ice crystals, and the supercooling of the system which may allow the formation of a glassy state and an even higher survival after cryopreservation.

During cryomicroscopy, a sudden change in opacity of the cytoplasm is often described as “blackening” or “flashing” (Acharya and Devireddy 2010; Day et al. 2000; Scheiwe and Korber 1984; Smith and Smiles 1953). The cause for blackening is not completely understood. It may occur as a result of light scattering from the surfaces of ice crystals (Körber et al. 1991; Smith 1961), from tiny gas bubbles formed in parallel (Steponkus and Dowgert 1981) or from rearrangement and aggregation of the intracellular organic matter (Dong et al. 2010; Stott and Karlsson 2009). Mazur et al. (2005) speculated that the darkening is the result of glass transition of intracellular water. In wheat pollen studied, a ‘darkening’ appeared before ice crystals were visible and indicate that ice crystals alone or combined with vitrification events may be the cause of this change in opacity in fresh and fast-dried pollen (Fig. 4a,b, Supplemental videos 1, 2). Interestingly, an abruptly second darkening termed ‘blackening’ or 'pollen turn black' occurred during warming. This phenomenon was also observed in mouse embryos and was suggested to be related with devitrification, but more likely with re-crystallization events of small intracellular crystals (Mazur et al. 2005). In general, cooling and warming rates are intertwined (Pegg 2007) and both processes affect the viability (Normah and Makeen 2008). During re-crystallization, ice crystals merge and damage the cells (Mazur 1984; Meryman 1966). However, there was a clear difference in the temperature at which the ‘darkening’ and ‘pollen turn black’ occurred between slow and fast cooling/warming and between fresh- and fast-dried pollen. Here, we speculate that at slower cooling/warming and at higher WCs, ice crystals had more time and volume to aggregate which facilitated an earlier ‘darkening’ and ‘blackening’ compared to fast cooling/warming and fast-drying. Nonetheless, these effects on the delay of ice-crystal formation did not favour a higher survival of the pollen grains. In further experiments, it has to be clarified if the ‘darkening’ and/or ‘blackening’ of cells is related with pollen viability changes and could be used to optimize the cryogenic procedure for wheat pollen preservation.

## Conclusion

Wheat is among the most important crops worldwide and the preservation of viable pollen would be beneficial for breeding programs. By analysing the change in pollen viability after exposure to different drying treatments, we observed that wheat pollen lost viability extremely fast (after > 12 min drying) and that the increased drying rate did not show the expected benefits. However, when fast-dried pollen was exposed to fast cooling/warming rates, we found some viable pollen, a reduction of the ice crystallization temperature, and a lower amount of ice crystals formed. Nevertheless, these results were not enough to achieve a high pollen survival. Long-term wheat pollen storage is still not efficient due to its extreme sensitivity to the removal of water molecules and osmotic concentration. Further investigations of the water properties of wheat pollen at high WCs, a better control of the ice crystallization and re-crystallization events during freezing and warming, and a better understanding of the vitrification of pollen at high water contents will help to develop more optimal cryopreservation protocols. Thereby, it should be considered that cooling/warming procedures should be extremely fast at WCs where viability is high and, eventually, cryoprotective chemicals, if entered, may stabilize the pollen without ice formation. These protocols and further testing in the fertilization of plants with cryopreserved pollen will elucidate if wheat pollen cryogenic storage can be an efficient tool for wheat conservation and breeding.

## Supplementary Information

Below is the link to the electronic supplementary material.

Supplementary file1 (MP4 66716 KB)

Supplementary file2 (MP4 61653 KB)

Supplementary file3 (MP4 117114 KB)

Supplementary file4 (MP4 109822 KB)

Supplementary file5 (MP4 72596 KB)

Supplementary file6 (MP4 78682 KB)

Supplementary file7 (DOCX 507 KB)

Supplementary file8 (PNG 56 KB)

Supplementary file9 (PNG 474 KB)

## Data Availability

The datasets generated during and/or analysed during the current study are available from the corresponding author on reasonable request.
