# Supplementary material for: Impact of drying and cooling rate on the survival of the desiccation-sensitive wheat pollen
Source: Plant Cell Rep. 2022 Jan 31;41(2):447–61. doi: 10.1007/s00299-021-02819-w (PMC8850252; doi:10.1007/s00299-021-02819-w)
Supplement: Supplementary file 7 — Supplementary file7 (DOCX 507 KB) [file 299_2021_2819_MOESM7_ESM.docx]

# Supplemental material


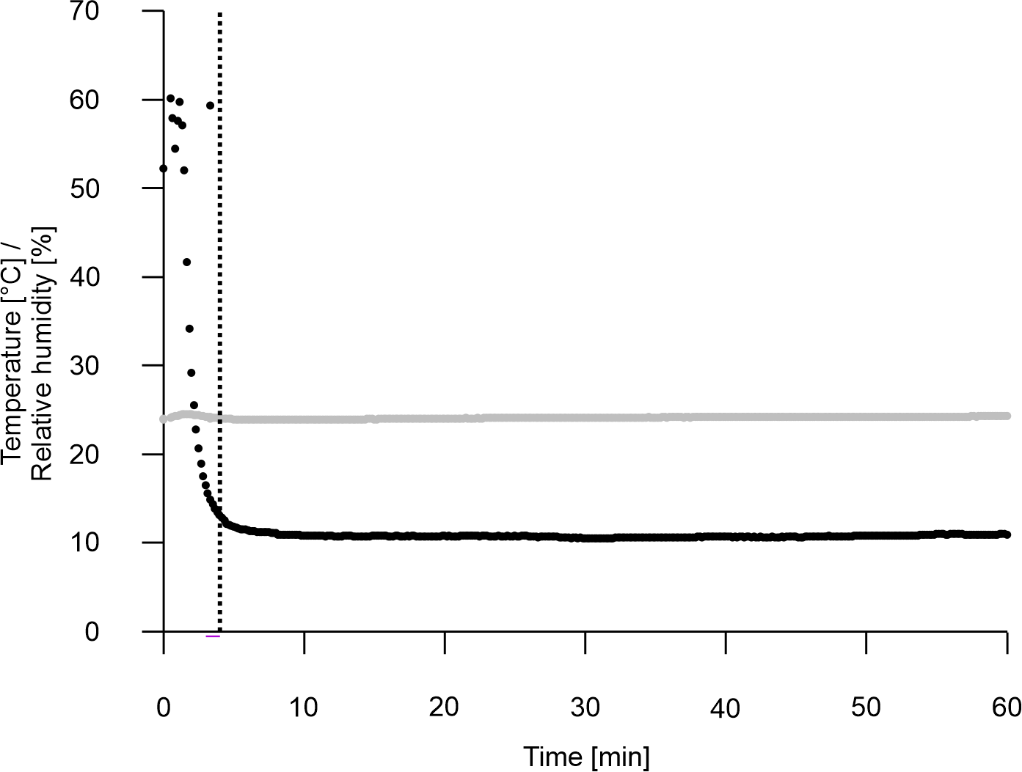


**Supplemental figure S1: Temperature (grey) and relative humidity (RH, black) were measured inside the pollen flash dryer using a data logger.** Temperature was held constant at 24.0 ± 1.0 °C. Equilibration of RH was achieved 4 minutes (vertical dashed line) after opening the flash drier to place pollen.


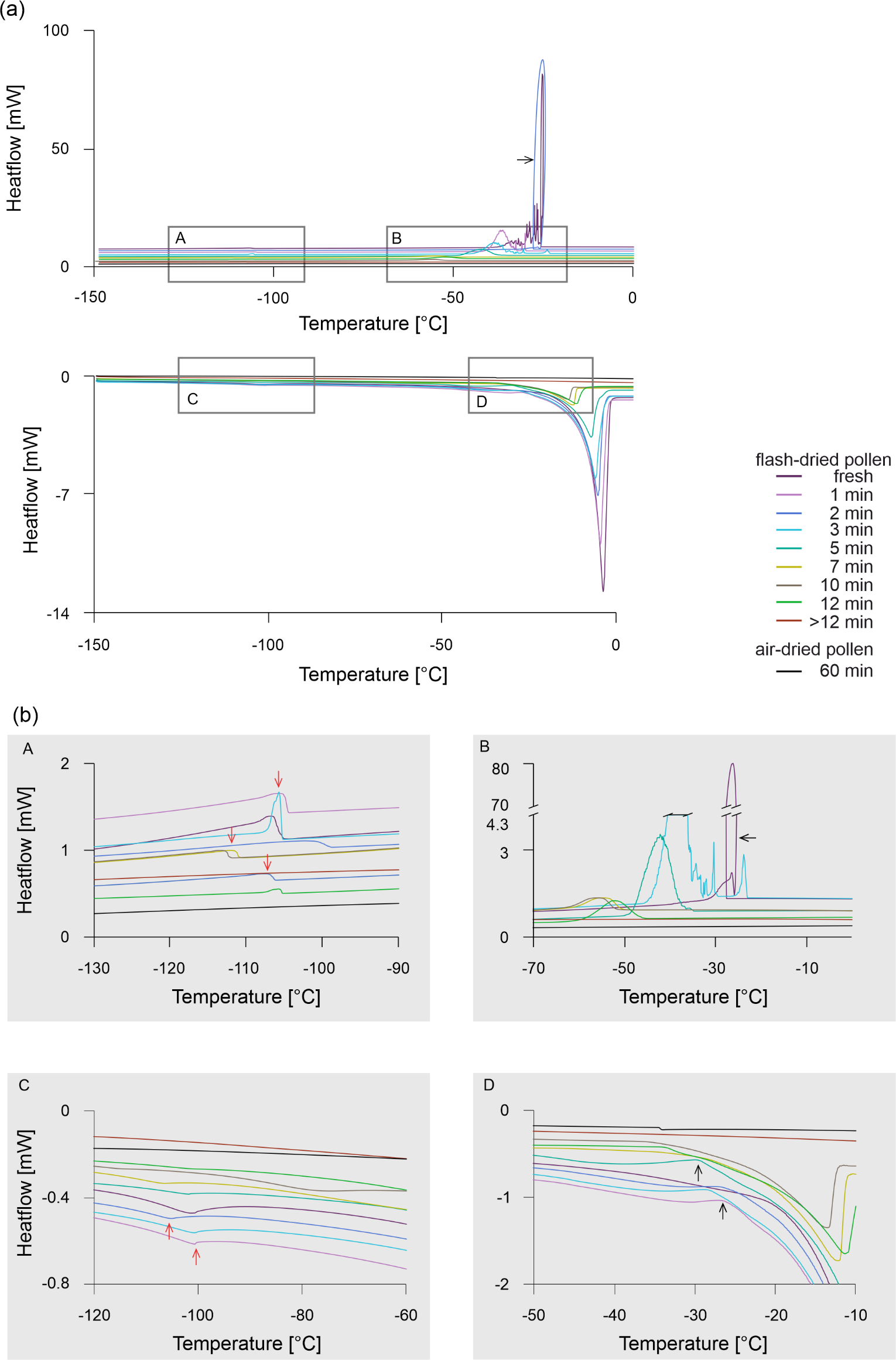


**Supplemental figure S2: Thermograms with enlarged sections show additional thermal transition events.** Pollen of wheat lines ‘Ferrum’ and TRI 9102 were used as fresh pollen or were air- or flash-dried for up to 60 minutes and heat flow measured using DSC. (a) Cooling and warming thermograms represent cooling and warming curves of selected replicates of pollen from wheat line ‘Ferrum’. (b) Enlarged sections in the cooling thermograms (A,B) point to eutectic formations (red arrows) and crystallization loop (black arrow) and broad crystallization peaks. Enlarged sections in the warming thermograms (C,D) show possible glass transitions (red arrows) and revitrifications (black arrows).

Supplemental video 1: Time-lapse video of fresh pollen of wheat line TRI 3633 during slow cooling with 1 °C min^-1^ cooling/warming rate.

Supplemental video 2: Time-lapse video pollen of wheat line TRI 3633 flash-dried for 5min during slow cooling with 1 °C min^-1^ cooling/warming rate.

Supplemental video 3: Time-lapse video of pollen of wheat line TRI 3633 air-dried for 60 min during slow cooling with 1 °C min^-1^ cooling/warming rate.

Supplemental video 4: Real-time video of fresh pollen of wheat line TRI 3633 during fast cooling with 150 °C min^-1^ cooling/warming rate.

Supplemental video 5: Time-lapse video of pollen of wheat line TRI 3633 flash-dried for 5min during fast cooling with 150 °C min^-1^ cooling/warming rate.

Supplemental video 6: Time-lapse video of fresh pollen of wheat line TRI 3633 air-dried for 60 min during fast cooling with 150 °C min^-1^ cooling/warming rate.
