# Supplementary figures and images for: Impact of drying and cooling rate on the survival of the desiccation-sensitive wheat pollen
Source: Plant Cell Rep. 2022 Jan 31;41(2):447–61. doi: 10.1007/s00299-021-02819-w (PMC8850252; doi:10.1007/s00299-021-02819-w)

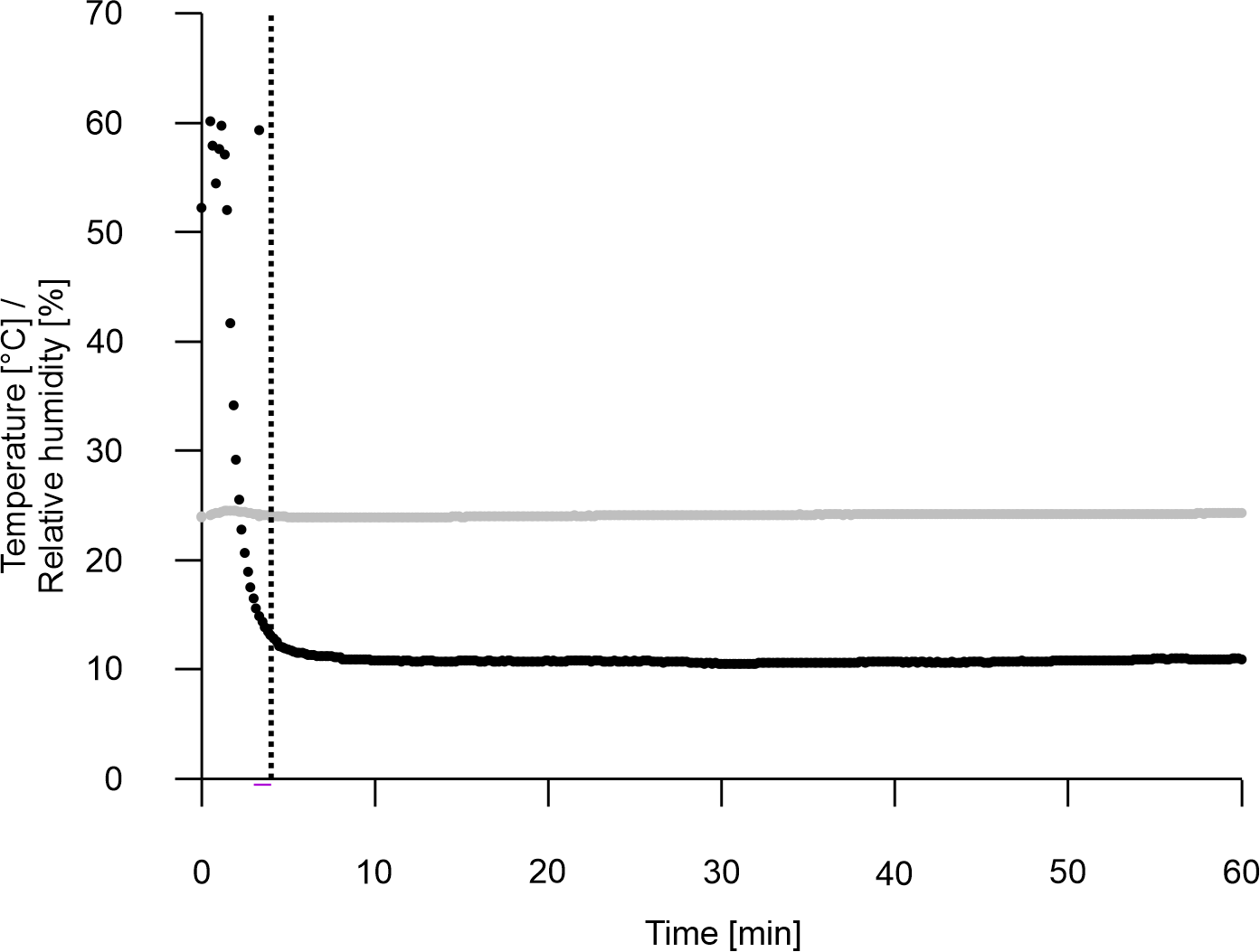

Supplement: Supplementary file 8 — Supplementary file8 (PNG 56 KB) [file 299_2021_2819_MOESM8_ESM.png]

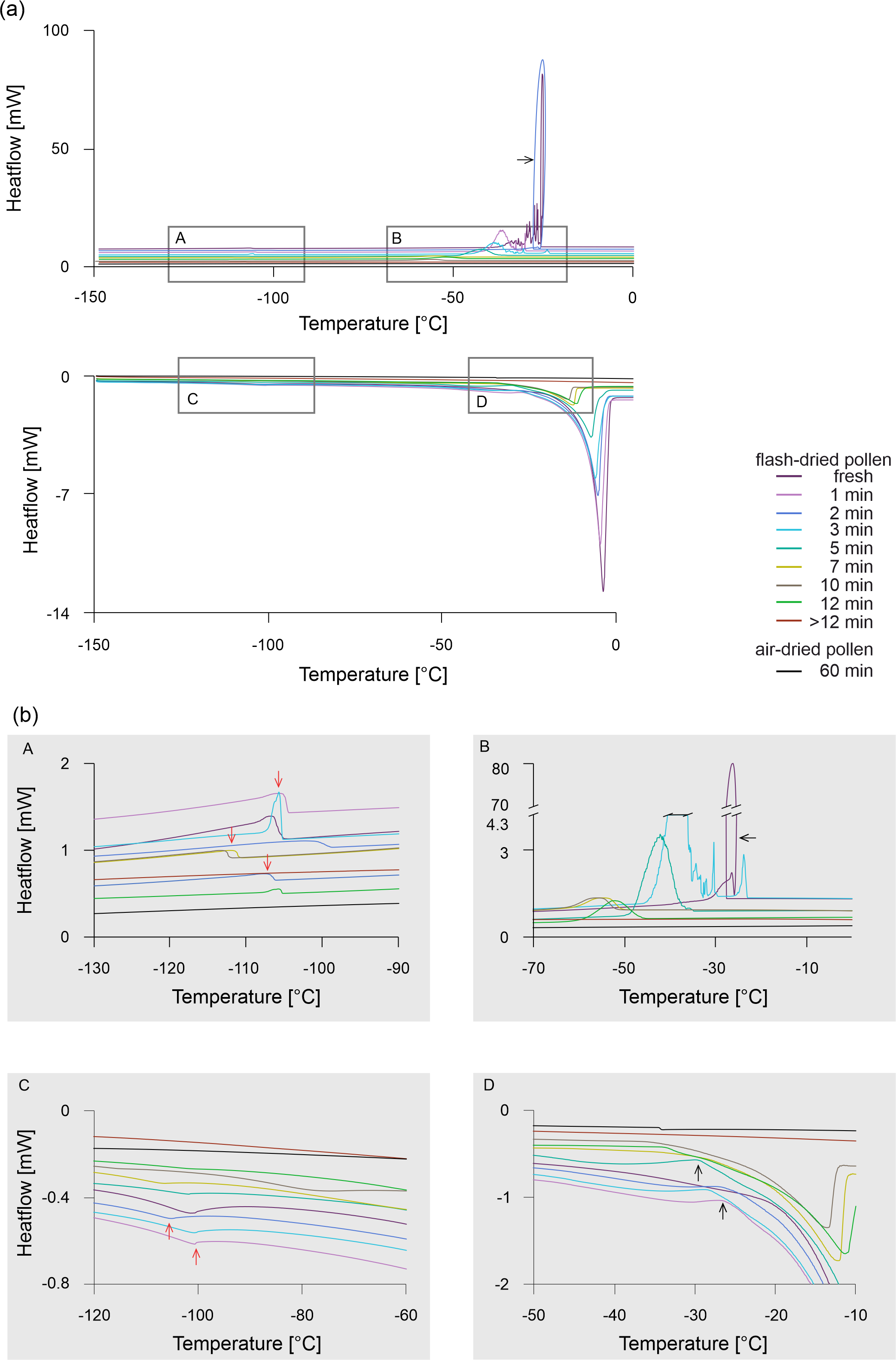

Supplement: Supplementary file 9 — Supplementary file9 (PNG 474 KB) [file 299_2021_2819_MOESM9_ESM.png]
